# Rat Extermination in Institutions

**Published:** 1916-02-26

**Authors:** 


### Rat Extermination in Institutions.

The Norway rat is so bold and enterprising in its search for a habitation, and withal so prolific and hardy, that it is quite possible for even new buildings to be infested; and for similar reasons practically all older hospitals harbour these ruffianly intruders. Of the various measures for abating this pest, in recent years none has claimed to be so successful as the means furnished by applied bacteriology, whereby infective disease can be propagated among rats (and mice) and their numbers enormously reduced in consequence. It is the *sine qua non* of such bacteriological remedies that they shall be non-poisonous to human beings and to the domestic animals, such as dogs, cats, fowls, and cattle; and it is highly desirable that they shall not harm such useful creatures as moles, insect-eating birds, nor such valuable ones as pheasants, partridges, hares, and other game animals. It is also very important that the disease from which the rats suffer shall not result in their flight when moribund to holes beneath floors, or indoors at all; for otherwise their decomposing corpses may prove a worse evil than even their presence when alive.

In Norway one of the best known of the viruses which fulfil these requirements is that known as Ratin, which has been on the market now for some years, and about which there is a general consensus of favourable opinion. This bacterial culture sets up among rats an epidemic disease of a very fatal character; the sufferers seek fresh air and water before their death takes place, so that they voluntarily evacuate their "runs" inside buildings and die in the open air. Probably institutions where rats have been troublesome have already availed themselves of this efficient and inexpensive preparation; but any hospital managers who have no experience of it may do well to obtain from the Ratin Laboratories, Windsor House, Kingsway, W.C., a copy of the official report on the recent clearance of rats in Aalborg, Denmark, effected by the use of Ratin. Never since the Pied Piper's days can such destruction in the rat colonies have been effected. The importance of this question for institutional managers is probably greater now than it has ever been because of the large numbers of temporary buildings, largely of wood construction, erected as military hospitals up and down the kingdom. Rats are known to be destructive as well as to be an essential element in the propagation of certain epidemic diseases (plague, for instance); and every means should be taken to eliminate them as far as is possible from institutions for the sick.

